# Supplementary material for: Chemical and Sensory Characterization of Xinomavro Red Wine Using Grapes from Protected Designations of Northern Greece
Source: Molecules. 2023 Jun 27;28(13):5016. doi: 10.3390/molecules28135016 (PMC10343362; doi:10.3390/molecules28135016)
Supplement: Supplementary file 1 [file molecules-28-05016-s001.zip › molecules-2420059-supplementary.pdf]

Table S1. Mean score of sensory attributes (± standard deviation) of Xinomavro red wines from six vineyards (Xin1–Xin6) in the Amyndeon PDO zone. Values with different lower case letters in the same row are significantly different according to the post-hoc test (α = 0.05).

	**Xin1**	**Xin2**	**Xin3**	**Xin4**	**Xin5**	**Xin6**	***p*-Value**
Aroma intensity	5.9±0.5 bc	5.8±0.4 bc	5.6±0.4 c	6.6±0.6 a	6.2±0.6 ab	5.8±0.3 bc	<0.0001
Cherry	2.7±0.5 cd	2.3±0.2 d	5.5±0.7 a	2.6±0.4 cd	4.1±0.9 b	3.0±0.8 c	<0.0001
Vanilla	0.1±0.2 bc	0.0±0.0 c	0.0±0.0 c	0.4±0.4 a	0.2±0.2 b	0.2±0.2 bc	<0.0001
Olive	1.1±0.2 c	0.0±0.0 d	3.0±0.4 a	2.9±1.1 ab	2.3±0.9 b	1.4±0.7 c	<0.0001
Sour cherry	1.4±0.4 bc	2.2±0.8 a	1.6±0.5 b	1.0±0.4 c	1.0±0.7 c	1.5±0.5 bc	<0.0001
Tomato	3.0±1.0 b	2.0±0.8 c	2.5±0.4 bc	3.9±0.8 a	4.5±0.9 a	4.6±1.2 a	<0.0001
Strawberry	6.3±0.5 a	5.5±0.9 b	4.1±0.2 c	4.2±0.2 c	5.4±0.2 b	5.1±0.2 b	<0.0001
Berry fruit	6.6±0.5 a	5.9±0.9 b	5.4±0.8 b	4.1±1,0 c	5.7±0.9 b	4.2±0.6 c	<0.0001
Mushroom	0.1±0.2 d	1.9±0.2 a	1.2±0.9 b	0.1±0.1 cd	0.1±0.1 d	0.7±0.5 bc	<0.0001
Green bell pepper	5.0±0.5 b	4.6±0.6 b	4.6±0.4 b	5.0±0.7 b	4.7±0.4 b	5.7±0.7 a	<0.0001
Plum	1.1±0.2 b	1.8±0.6 a	1.4±0.5 ab	1.0±0.6 b	1.0±0.6 b	1.1±0.4 b	<0.0001
Spices	5.1±0.4 b	5.9±0.5 a	6.1±0.8 a	6.0±0.5 a	5.0±0.7 b	5.6±0.4 ab	<0.0001

Table S2. Mean values (± standard deviation, n = 3) of enological parameters of Xinomavro musts at technological maturity from six vineyards (Xin1–Xin6) in the Amyndeon PDO zone.

	Xin1	Xin2	Xin3	Xin4	Xin5	Xin6
Brix	22.5±0.1	22.1±0.0	23.1±0.1	23.5±0.1	22.7±0.1	23.3±0.2
pH	3.19±0.02	3.13±0.01	3.11±0.01	3.16±0.02	3.18±0.00	3.17±0.00
Titratable acidity (tartaric acid g/L)	8.6±0.0	9.4±0.1	9.1±0.2	9.2±0.3	9.2±0.2	8.8±0.0

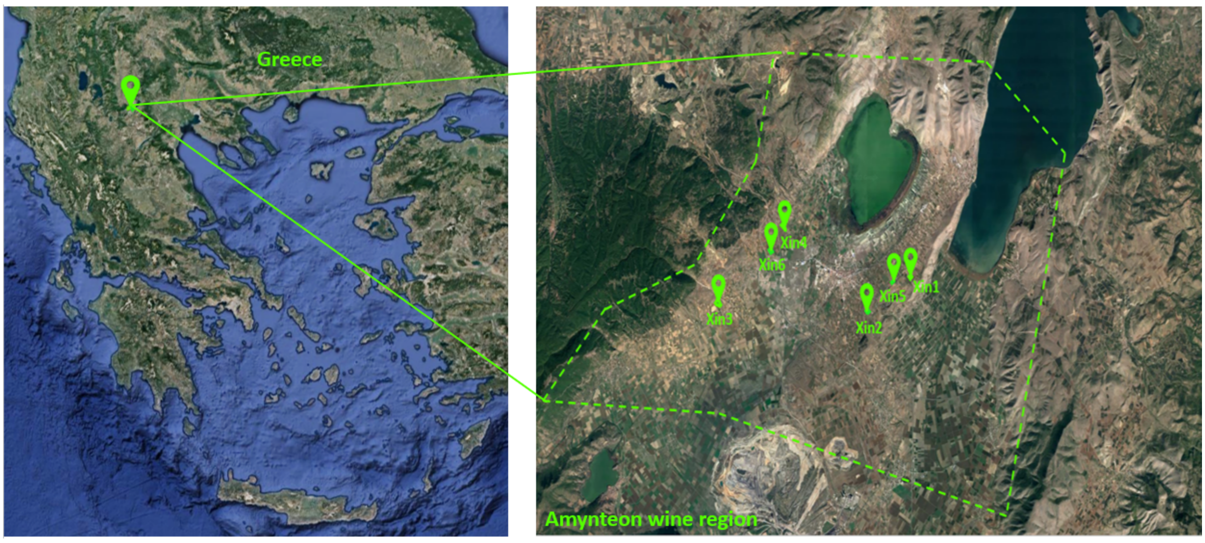

Figure S1. Location of the study area (Amyndeon PDO wine region) and vineyards with their codes (Xin1–Xin6).
